# Effectiveness of 10 polymorphic microsatellite markers for parentage and pedigree analysis in plateau pika *(Ochotona curzoniae)*

**DOI:** 10.1186/1471-2156-11-101

**Published:** 2010-11-10

**Authors:** Kexin Li, Jianing Geng, Jiapeng Qu, Yanming Zhang, Songnian Hu

**Affiliations:** 1 Key Laboratory of Adaptation and Evolution of Plateau Biota, Northwest Institute of Plateau Biology, the Chinese Academy of Sciences, 59 XiGuan Avenue, Xining, Qinghai, 810001, PR China; 2 Key laboratory of Genome Information and Sciences, Beijing Institute of Genomics, the Chinese Academy of Sciences, No.7 Beitucheng West Road, ChaoYang District, Beijing, 100029, PR China; 3 Graduate University of the Chinese Academy of Sciences, Beijing 100049, PR China

## Abstract

**Background:**

The plateau pika *(Ochotona curzoniae) *is an underground-dwelling mammal, native to the Tibetan plateau of China. A set of 10 polymorphic microsatellite loci has been developed earlier. Its reliability for parentage assignment has been tested in a plateau pika population. Two family groups with a known pedigree were used to validate the power of this set of markers.

**Results:**

The error in parentage assignment using a combination of these 10 loci was very low as indicated by their power of discrimination (0.803 - 0.932), power of exclusion (0.351 - 0.887), and an effectiveness of the combined probability of exclusion in parentage assignment of 99.999%.

**Conclusion:**

All the offspring of a family could be assigned to their biological mother; and their father or relatives could also be identified. This set of markers therefore provides a powerful and efficient tool for parentage assignment and other population analyses in the plateau pika.

## Background

Plateau pikas *(Ochotona curzoniae) *are small lagomorphs that inhabit the high alpine grasslands of the Tibetan plateau of China. They live in cohesive families and occupy burrow systems. Plateau pikas exhibit monogamy, polygyny, polyandry and promiscuous mating systems [1]. Approximately 57.8% of pikas exhibit philopatry, and dispersal movements are extremely restricted, although some dispersal may occur to ensure spatial separation of kin that may otherwise mate [2]. Inbreeding would be expected to occur under these circumstances. Dominant males monopolize mating in order to maximise reproductive fitness and minimise inbreeding depression. Previous methods to determine the level of inbreeding and how it affects the population depended mainly on direct observation due to the lack of molecular tools. Although family group behaviors have been described through observation in the plateau pika [2-5], details of family structures lack corroborative molecular evidence. In some breeding systems such as lekking, polygyny, polyandry and cooperative breeding, it may be impossible to determine parentage from direct observations [6]. Therefore, molecular tools such as microsatellites markers are necessary to obtain genetic information about family structure, social behavior and dispersal. Microsatellite markers, also called short tandem repeats (STRs), are ideal molecular markers for various genetic studies because they are highly polymorphic, codominant in the manner of inheritance, and easy to genotype by using the polymerase chain reaction (PCR) [7]. Previous studies have demonstrated the ability of microsatellite markers to determine parentage [8,9], analyze pedigree [10] and identify species [11] using species-specific STRs. The success rates of parentage assignment in some studies are high, for example, assignment success for a hatchery population of brown sole [12] and Chinese shrimp [9] reached 92.2% and 92.9%, respectively.

In this study, we evaluated the reliability of our previously developed 10 microsatellite loci [13] for parentage determination and discuss their use for future population genetic analyses of the plateau pika populations.

## Results

**Figure 1:**
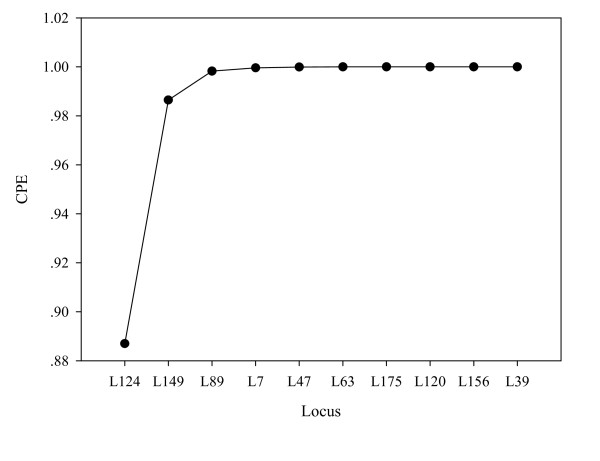
Combined probability of exclusion increasing with number of loci.

The mean proportion of individuals genotyped was 0.9556 and expected heterozygosity (He) ranges from 0.784 to 0.905, while PIC ranged from 0.623 to 0.857. The number of alleles (k) per locus ranged from 5 to 11. The PE for individual loci was from 0.351 to 0.887, and the CPE for the 10 loci used in this study was as high as 99.999% (*Table *1). Moreover, we find that the CPE for a core set of 5 loci in parentage determination reached 99.99% (*Table *2) and the CPE increased much slowly with each additional locus (*Figure *1). Combined non-exclusion probabilities for the first parent, second parent and parent pairs are 2.28E-03, 4.9E-05 and 5E-08, respectively.

**Table 1:** Parameters of the 10 microsatellite loci

Locus	k	N	Hobs	Hexp	PIC	NE-1P	NE-2P	NE-PP	F(null)	NE-I	NE-SI	HW	PD	PE	CPE
EU518185	7	15	0.933	0.834	0.779	0.560	0.383	0.203	-0.076	0.047	0.349	NS	0.895	0.887	0.887
EU518186	11	14	0.857	0.884	0.836	0.452	0.290	0.121	0.000	0.083	0.382	**	0.927	0.880	0.98644
EU518196	11	13	0.846	0.905	0.857	0.413	0.258	0.100	0.002	0.073	0.372	NS	0.906	0.872	0.9982643
EU518194	10	16	0.938	0.875	0.83	0.466	0.301	0.131	-0.050	0.104	0.415	NS	0.932	0.773	0.999606
EU518192	8	14	0.857	0.831	0.775	0.560	0.383	0.198	-0.035	0.021	0.309	NS	0.900	0.760	0.9999054
EU518193	8	15	0.933	0.784	0.723	0.630	0.452	0.262	-0.111	0.137	0.435	NS	0.828	0.745	0.9999759
EU518189	6	16	0.938	0.823	0.767	0.578	0.400	0.218	-0.084	0.057	0.356	NS	0.877	0.662	0.9999918
EU518184	5	16	0.688	0.690	0.623	0.743	0.569	0.383	-0.041	0.037	0.335	NS	0.827	0.463	0.9999956
EU518187	8	14	0.714	0.810	0.753	0.588	0.409	0.218	0.058	0.052	0.351	NS	0.907	0.437	0.9999975
EU518191	8	15	0.600	0.809	0.752	0.593	0.415	0.228	0.143	0.087	0.39	NS	0.803	0.351	0.9999984

Locus: Named from GenBank accession; k: Number of alleles at the locus; N: Number of individuals typed at the locus; Hobs: Observed heterozygosity; Hexp: Expected heterozygosity; PIC: Polymorphic information content; NE-1P: Average non-exclusion probability for one candidate parent; NE-2P: Average non-exclusion probability for one candidate parent given the genotype of a known parent of the opposite sex; NE-PP: Average non-exclusion probability for a candidate parent pair; F(Null): Estimated null allele frequency; PD: Power of discrimination; PE: Power of exclusion; CPE: Combined probability of exclusion; NS = not significant, ** = significant at the 1% level; NE-I: Average non-exclusion probability for identity of two unrelated individuals; NE-SI: Average non-exclusion probability for identity of two siblings.

**Table 2:** Parentage assignment of two families' embryos by 10 microsatellite loci

Offspring	Candidate mother or father	Pair loci number	Pair loci mismatching	Pair LOD score	Pair Delta	Pair confidence
p8-1	pF8	10	0	2.21E+00	2.21E+00	*
p8-2	pF8	10	0	3.86E+00	3.86E+00	*
p8-3	pF8	9	0	3.04E+00	3.04E+00	*
p10-1	pF10	10	0	6.97E+00	6.97E+00	*
p10-2	pF10	10	0	4.29E+00	4.29E+00	*
p8-1	pM8	10	0	5.88E+00	5.88E+00	*
p8-2	pM8	10	1	2.26E+00	2.26E+00	*
p8-3	pM8	9	0	3.60E+00	3.60E+00	*
p10-1	pM12	8	4	-1.55E+01	0.00E+00	
p10-2	pM12	8	4	-1.55E+01	0.00E+00	

*: for strict confidence. If the candidate parent is not the most likely, this column will be blank.

Pair LOD score: Log-likelihood ratio for a parent-offspring relationship between the first candidate parent and the offspring.

For the family-8, LOD values for maternity were from 2.21 to 3.86, which indicates that all offspring are correctly assigned to their mother, pF8, and these results are consistent with pedigree information. LOD values for the candidate paternity of family-8 ranges from 2.26 to 5.88 and their assignment to their father, pM8, is consistent with observations in the field (*Table *2). Nevertheless, there is a mismatched locus between p8-2 and pM8, namely EU518189. Similarity of two individuals based on allele sharing of the 10 loci and the proportion of shared alleles between individuals was calculated (*Table *3).

**Table 3:** Similarity of two individuals based on allele sharing

	pF3	pF8	pF10	pM10	pM13	pM14	pM15	pM2	pM4	pM6	pM7	pM8	pM12
p8-1	0.2	0.55	0.4	0.5	0.2	0.22	0.17	0.1	0.3	0.31	0.3	0.65	0.19
p8-2	0.2	0.55	0.3	0.28	0.2	0.17	0.17	0.1	0.15	0.25	0.25	0.6	0.06
p8-3	0.28	0.61	0.28	0.25	0.22	0.25	0.19	0.17	0.33	0.29	0.44	0.67	0.14
p10-1	0.15	0.3	0.65	0.17	0.25	0.33	0.28	0.15	0.3	0.19	0.15	0.2	0.31
p10-2	0.3	0.35	0.6	0.33	0.3	0.28	0.33	0.3	0.3	0.31	0.25	0.25	0.25

For the family-10, LOD values for maternity were 4.29 and 6.27 and the offspring could be correctly assigned to their biological mother, pF10 (*Table *2). The proportion of shared alleles between pF10 and offspring was 0.60 and 0.65 (*Table *3). None of the offspring from either family could be assigned to pF3. However, LOD values for paternity of family-10 are below 0. Proportion of shared alleles between p10-1, p10-2 and pM12 are 0.25 and 0.31, respectively. Allele sharing proportion between offspring from family-10 with pM10 is nearly equal to that with pM12 (*Table *3), with an average of 0.28 versus 0.25, but both are below 0.5. This indicates that the genetic distance between family-10 and pM10 is smaller than that between family-10 and pM12. Thus, the paternity for the embryos in family-10 could not be conclusively attributed to either pM12 or pM10.

## Discussion

The use of microsatellite genotyping for parentage assignment and population genetics studies is a common procedure in wild animals. He and PIC values presented here for a set of microsatellites are high compared to similar studies in other species [14]. Paternity exclusion is a commonly used method for parentage determination. The CPE for our set of 10 microsatellite loci is over 99.99% (*Table *1). The CPE value for this set of microsatellite markers is higher than that of 15 microsatellites used in the horse for pedigree verification [15]. Figure 1 shows that the combined probability of exclusion is already quite high for a small number of markers. Although it is not yet established whether all the 10 loci used here are autosomal, their application for the purpose of parentage assignment appears to be satisfactory, especially when data from all 10 is analysed together, as was done here.

As demonstrated in this study, all the offspring could be assigned to their true mother and the paternity for family-8 was satisfactorily established, except for a mismatch between embryo, p8-2 and the male pM8. This is probably due to a mutation or a null allele in locus EU518189. For the family-10, a father could not be identified from the candidate fathers. The members from family-10 were genotyped three times to insure veracity of the methods used. There are still 4 loci with genotype mismatches in the paternity-offspring pairs. A negative LOD score means that the candidate parent is not likely to be the true parent. This could be because the true father is probably not present in the candidate pool. The exclusion analysis shows that pM12 is closely related to family-10 with half of the total loci matching, which makes him more likely to be their father than pM10, and this is inconsistent with the observation that pM10 was captured while moving within the family-10 burrow. Furthermore, the allele sharing matrix between embryos from family-10 and pM10 shows that pM10 and pM12 are both closely related with family-10. Taken together, these results demonstrate that pM10 and pM12 may be close relatives of p10-1 and p10-2. This indicates that the set of markers show great potential in not only parentage assignment, but also in determining close relatives. Although pM10 was captured along with family-10, it does not appear to be the biological father of the offspring analyzed here. We conclude that there are other males living in a family, and that philopatry exists in social plateau pikas which is consistent with former observation. In this study, pM12 showed significant genetic similarity to family-10, but wasn't captured within the family-10 home range. This supports Dobson's observation that there exists dispersal in this species [2].

## Conclusions

In conclusion, we have developed a set of 10 microsatellite markers that has proved to be a powerful tool for parentage verification and individual identification of plateau pikas from the Qinghai-Xizang (Tibetan) plateau of China.

## Methods

### Sample collections and molecular techniques

**Figure 2:**
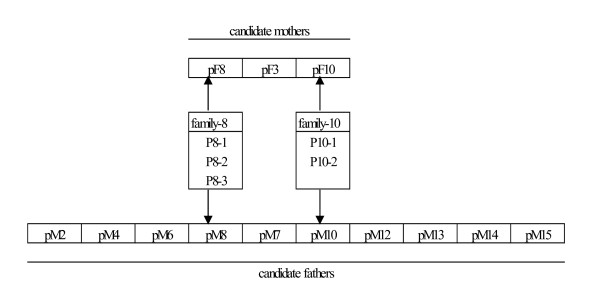
Relationship between all the individuals by direct field observations when sample collecting.

Plateau pikas were trapped by the string-noose method [16] in the southeastern region of the Tibetan Plateau, about 17 km east of Dawu town (34°24′N, 100°21′E, at an elevation of 3846 m), Maqin County, Qinghai Province, People's Republic of China. This study was approved by China Zoological Society. The captured adult pikas were euthanized humanely by experienced laboratory assistant by cervical dislocation as per the recommendations by the China Wildlife Conservation Association. The animals were dissected and muscle tissue samples were obtained. Pregnant females were anesthetized before dissection. The muscle and embryo tissues were collected and stored in 95% ethanol. Animals from two families, designated family-8 and family-10 were analyzed. Tissue samples obtained from three embryos (p8-1, p8-2, p8-3) from family-8; and two embryos (p10-1, p10-2) from family-10, were from the females, pF8 and pF10, respectively. Based on direct observations, the corresponding probable fathers of these embryos were the males, pM8, pM10. Eight other males from the area were randomly collected and used in the analysis (*Figure *2. Another female, pF3 was also included as an additional candidate mother.

All the sample tissues were washed with distilled water to remove the ethanol from the surface before use in DNA extraction. Genomic DNA was isolated from the muscle tissues of the parents and their progeny using the standard phenol-chloroform extraction protocol. Negative controls were included to confirm that no contamination had occurred during the extraction procedure. The 10 pairs of microsatellite primers employed here [EU518185, EU518186, EU518196, EU518194, EU518192, EU518193, EU518189, EU518184, EU518187, EU518191] have been described in our earlier work [13,17]. These 10 loci selected for this study were chosen based on their ease of amplification, particularly when analyzing impure or trace samples that occur frequently in wildlife studies, while the other 3 loci [EU518195, EU518188, EU51819] described in our previous study require a high quality of genomic DNA that might be difficult to obtain in some studies. PCR was carried out in a total volume of 10 μL containing approximately 10 ng of genomic DNA, 1 pmol unlabelled reverse primer, 1 pmol fluorescently labeled forward primer and Taq DNA polymerase (TakaRa), in an appropriate buffer. The other reactions conditions were as follows: an initial denaturation at 94°C for 5 min, followed by 10 cycles of 94°C for 30 s and 72°C for 30 s, and then 20 cycles of 89°C for 30 s and 72°C for 30 s, with a final extension at 72°C for 20 min [12,14]. Appropriate controls were included for each set of PCR reactions to ascertain that the template and the primers were not contaminated. PCR products were purified using ethanol-ammonium acetate. Genotyping was based on fragment length polymorphism of the fluorescently tagged DNA fragments using the 3730 DNA Analyzer with GeneScan™-500 LIZ™ Size Standard and GeneMarkerV1.75 software (all from Applied Biosystems, Inc.).

### Genetic parentage analysis

The allelic size data set was checked for numeric errors and null alleles at 95% confidence interval using MICRO-CHECKER [18]. Hardy-Weinberg equilibrium was tested in GENEPOP version 3.4 [19]. Cervus 3.0 [20] was used for the analysis of all the following parameters: parentage analysis by calculating logarithm of odds (LOD) scores given by the natural logarithm of the overall likelihood ratio, homozygotes (Ho), heterozygotes (He), power of discrimination (PD), polymorphism information content (PIC), average non-exclusion probability for one candidate parent (NE-1P), average non-exclusion probability for one candidate parent given the genotype of a known parent of the opposite sex (NE-2P); average non-exclusion probability for a candidate parent pair (NE-PP), estimated null allele frequency F (Null), average non-exclusion probability for identity of two unrelated individuals (NE-I), average non-exclusion probability for identity of two siblings (NE-SI) [20]. Power of exclusion and combined power of exclusion of the 10 loci were calculated from allele frequencies by PowerStats V1.2 (Promega Corporation, U.S.A.). Furthermore, distances between pairs of individuals based on allele sharing were calculated using Microsatellite Tookit http://www.animalgenomics.ucd.ie/sdepark/ms-toolkit/.

## Authors' contributions

KXL performed all the molecular experiments, analyzed all the data set, drafted manuscript, make the Figure 1, Figure 2, Table 1, made revisions for this manuscript. JNG made table 2 and table 3 and described them. JPQ collected the samples. YMZ took response for all this paper. SNH directed all the experiment and revised this manuscript. All authors read and approved the final manuscript.
